# Reducing risks for infant mortality in the Midlands, UK: a qualitative study identifying areas for improvement in the delivery of key public health messages in the perinatal period

**DOI:** 10.1186/s12884-022-05092-1

**Published:** 2022-10-17

**Authors:** Thillagavathie Pillay, Jane Feeney, Claire Walters, Hollie Nelson, Lucy Thomas, Dawn Lewis, Karen Anderson, Anjali Petkar

**Affiliations:** 1 Neonatal Unit, University Hospitals Leicester NHS Trust, Kensington Building, Leicester Royal Infirmary, LE15WW Leicester, United Kingdom; 2 Faculty of Science and Engineering, University of Wolverhampton, Wolfruna Street, WV1 1LY Wolverhampton, United Kingdom; 3 College of Life Sciences, University of Leicester, University of Leicester, University Road, LE1 7RH Leicester, United Kingdom; 4 Neonatal Unit, The Dudley Group of Hospitals NHS Trust, Russells Hall Hospital, Pensnett Road, DY1 2HQ Dudley, West Midlands United Kingdom

**Keywords:** Parent education, Parent empowerment, Infant mortality, Reducing risks, Neonatal public health

## Abstract

**Background:**

The Midlands has amongst the highest rates of neonatal and infant mortality in the UK. A public health parent education and empowerment programme, aimed at reducing key risks associated with this mortality was established and evaluated in the region. This was undertaken in an attempt to identify areas for optimal delivery of the public health messages around reducing risks for neonatal and infant mortality.

**Method:**

Qualitatively assessment, using the software package Dedoose®, was undertaken. This involved analysis of reflections by the programme trainers, after the delivery of their training sessions to parents, families and carers, between 01 January and 31 December 2021. These were intended to capture insights from the trainers on parent, family, carer and staff perspectives, perceptions/misperceptions around reducing risks for infant mortality. Potential areas for improvement in delivery of the programme were identified from this analysis.

**Results:**

A total of 323 programmes, comprising 524 parents, family members and carers were offered the programme. Analysis of 167 reflections around these interactions and those of staff (n = 29) are reported. The programme was positively received across parents, families, carers and staff. Four overall themes were identified: (a) reach and inclusion, (b) knowledge, (c) practical and emotional support and (d) challenges for delivery of the programme. Recommendations for improved delivery of the programme were identified, based on qualitative analysis.

**Conclusion:**

This novel approach to empowerment and education around neonatal public health messaging is a valuable tool for parents, families, carers and staff in the Midlands. Key practical recommendations for enhancing delivery of these critical public health messages were identified from this qualitative research. These are likely to be of value in other parts of the UK and globally.

## Introduction

Infant mortality is a key indicator of the health of a nation. In the United Kingdom (UK), the vast majority of infant mortality is due to deaths within the neonatal period. Infant mortality ranges from 1.7 deaths per 1000 live births in some parts of the UK, to a high of almost 7 deaths (6.8) per 1000 live births in the Midlands [1]. The Midlands has notoriously held high rates of infant mortality, and there have been numerous reports presented to cabinet and published outlining the key associations with infant mortality in the region. These include smoking, lack of breast feeding, prematurity, low birth weight, smoking, extremes of maternal age at delivery, domestic abuse, congenital anomalies, ethnicity, social deprivation, lack of maternal education, sudden infant death syndrome and infection [2–4].

Intense child death overview panels, local and regional perinatal mortality reviews, National Mothers and Babies confidential enquiries (MBRRACE Reducing Risk through Audits and Confidential Enquiries) and other supportive agencies addressing potentially avoidable factors relating to medical and social support are regularly and intensely conducted in England. However there has been no analysis of population perspectives (parents, families and carers of those with new babies or those expecting a new baby) around risks associated with infant mortality, and the impact of their carer education and empowerment in the antenatal and postnatal period, in understanding and modifying behaviours in response to their knowledge of these risks.

Our objective was to qualitatively assess perspectives on education and empowerment around risks for infant mortality in the region in families that were expecting or had just delivered a new baby, so that it could (a) provide a base for local knowledge, (b) inform how better to deliver our messages, (c) and over time, define which aspects to focus on moving forward that would best benefit the local population. We report here on qualitative analysis of the perspectives shared by parents, families and carers, through reflections from trainers within a neonatal public health education and empowerment Programme (STORK Programme) in a local district general hospital in The Midlands.

### Background of the local neonatal public health programme

In stretching the boundaries of perinatal care, a public health programme, aimed at reducing the risks associated with infant mortality through education and empowerment to families in parts of the Midlands began in 2017 [5, 6]. This programme [7] (STORK for parents, families and carers) addresses key public health messaging around chief preventable risks associated with infant mortality comprises (a) basic bystander life support education, (b) how to deal with a choking child, (c) recognising signs of illness in baby, (d) safe sleeping advice, (e) breast feeding support and advice, (f) understanding the risks associated with smoking in and after pregnancy, around baby, and smoking cessation advice, (g) managing a crying baby, and signposting to healthy lifestyle and perinatal mental health support in relevant participating regions. Delivery of ‘one’ programme to a parent, family or carer means education on all aspects a) to g) above, with specific focus on areas of greatest need.

Its focus is to highlight key risks and strategies to reduce the risks around infant mortality in the region, using modern aids such as mobile applications, and linking in to NHS and other relevant online resources. Parents and families with new born babies or those expectant parents, are taught the key components of the programme, using a mobile application [8] and in so doing are informed around risks associated with infant mortality in the region. The programme is modified in participating units, as per available resources, to either offer individualised 1:1 learning with families, group or signposting sessions. STORK is the acronym for Supportive Training Offering Reassurance and Knowledge, and parent feedback on the programme has previously been published. For sessions of education of STORK programme education with parents, family members or carers, staff members were routinely encouraged to be observers. This was part of the overall delivery of the STORK programme at the hospital, to promote awareness amongst staff so that they could better support the parents, family members and carers whilst in hospital. Multiple sessions with parents, families and carers to ensure all aspects were delivered comprise delivery of ‘one programme’. This included follow up conversations with parents, families and carers after discharge from the neonatal unit.

Anchored at the University of Wolverhampton, Research Institute and Faculty of Science and Engineering, it has been supported in participating local regions variably by individual NHS Trusts, in which the programme has been embedded into discharge services for neonatal units, or through Public Health, Local Maternity and Neonatal Systems and City Councils. The programme focuses on parent, family and carer education and empowerment, utilising NHS and other resources. Its strength lies in the collation of material into a single platform around reducing risks for infant mortality, in a user-friendly mobile application, 1:1 parent, family and carer training, and delivered by support workers in this district general hospital. Its success with parents has been previously described from another local district hospital [5]. Here, in a survey of 218 parents or carers in Wolverhampton, 215 (98.6%) indicated it instilled confidence in them caring for their babies after discharge from the neonatal unit, that they would recommend the programme to others, and that the programme was useful. The remaining three did not comment as English was not their first language. Free text feedback from parents included amongst other comments, that ‘it was a great programme’, that ‘all parents should be offered this’, ‘. and we learnt a lot of what we didn’t know’. The intention of the programme is to, over time, work towards behavioural changes that cascade multi-directionally, to friends and relatives, subsequent children, parents and grandparents and carers.

## Methods

### Setting

This project was undertaken at a Dudley Group of Hospitals NHS Trust, local district general hospital catering for approximately 4100 births per year in the Midlands. With the support of its Nurture and Resilience Steering Group, local Council, its local department of Public Health funded two part-time posts for support workers to work within the hospital’s neonatal in-patient setting, providing 1:1 education and empowerment for parents whose babies were admitted to its neonatal services. Additional support where needed for vulnerable families in the paediatric and maternity setting was offered, in delivering the messages around reducing the risks for infant mortality. Vulnerable families included those with a previous neonatal or infant death, recent immigrants or refugees to the UK, mothers under 18 years of age who had declined the family nurse partnership established to support teenage pregnancies, those mothers with learning difficulties, were smokers in pregnancy, experiences substance misuse, domestic violence, mental health issues, where there were safeguarding concerns, or concerns raised by health care professionals during pregnancy or after birth about parenting capabilities such as complex social lives or unsafe sleep practices. Approximately a third of the mothers and families who received the STORK programme in our hospital were classified as vulnerable, and a quarter, ethnic minority.

The support workers (STORK Trainers) were employed at the equivalent grade of band 4 nurses at the Trust. They had healthcare backgrounds, were educated to a minimum of degree level, and had previous experience in data capture in research. They were all educators prior to employment with the Hospital and had hands-on experience in patient counselling. A one day STORK programme-specific full day training was delivered at induction into the post, by the developer of the programme (TP) and supported by training for its individual components which are referenced in the app [8] and based on online NHS material for safe sleep, recognising signs of illness, and smoking cessation. Online training resources from the national centre for smoking cessation were utilised. Bystander life support and choking training were based on the algorithm supported by our BLISS our national charity for babies born preterm and ill [9], and St Johns Ambulance guidelines. A 2 day maternity-led breastfeeding training session, supporting the Unicef Baby Friendly Initiative [10], for which the hospital’s maternity services has received full accreditation, was also undertaken. Training to manage the crying baby (ICON) [11] was also included. All of these are referenced in the mobile app. STORK trainers observed an established STORK trainer for around 4 weeks before actively starting own teaching sessions.

### Data collection

Prior to commencement of the project the trainers were given direction on documentation of reflections around their work, as and when they considered it appropriate, i.e. while in hospital or after multiple sessions with parents, families and carers.The structure of the reflection was free text, intended to capture the following broad themes: (a) what did you learn from your engagement with the parents/families/carers/staff, i.e. what did parents generally know (b) what went well, (c) what could have been done differently, or better. Qualitative data were collected in the form of written reflections and voice memos by the STORK trainers teaching the reducing the risks associated with infant mortality messaging to parents, families and carers. Three trainers (occupying two posts) recorded their reflections following sessions they conducted with participants of the STORK programme between January and December 2021.

All participant data was anonymised. All data remained confidential. The anonymity of the parents, families and carers who participated was maintained. No identifying information on participants was shared. The written reflections were typed up by the trainers and the voice memos were later transcribed using automated transcription software and edited for accuracy.

We selected trainer reflections as the most robust way of capturing perspectives for this study. We considered the option of selecting parents for interview, but felt that greater value could be obtained through larger scale reflections, across a wider mix of patients, and suited our objective better.

### Data analysis

The reflections (written notes and transcription files) were uploaded to Dedoose®, a software application for analysing qualitative and mixed methods research. An experienced social scientist coded the reflections using thematic analysis during two stages [12]. First, we generated initial codes. A second phase of coding was then undertaken involving organising the codes into overall themes and a process of collaboration with the principal researcher, who was a clinician (consultant neonatologist). A combination of inductive and deductive coding was carried out in two stages. Utility of a clinician with experience in the development of the programme and in parent counselling, together with an experienced social scientist was designed to ensure optimal derivation of codes and themes.

First, overall categories were identified before the data analysis process: baseline knowledge around basic life support and choking training and the shaken baby (ICON https://iconcope.org/ [9]); and cultural perceptions around safe sleep, breastfeeding, artificial feed and smoking. The data were analysed line-by-line and codes were added. The list of codes was then organised and refined, resulting in an initial list of categories and codes (Table 1).

**Fig. 1:**
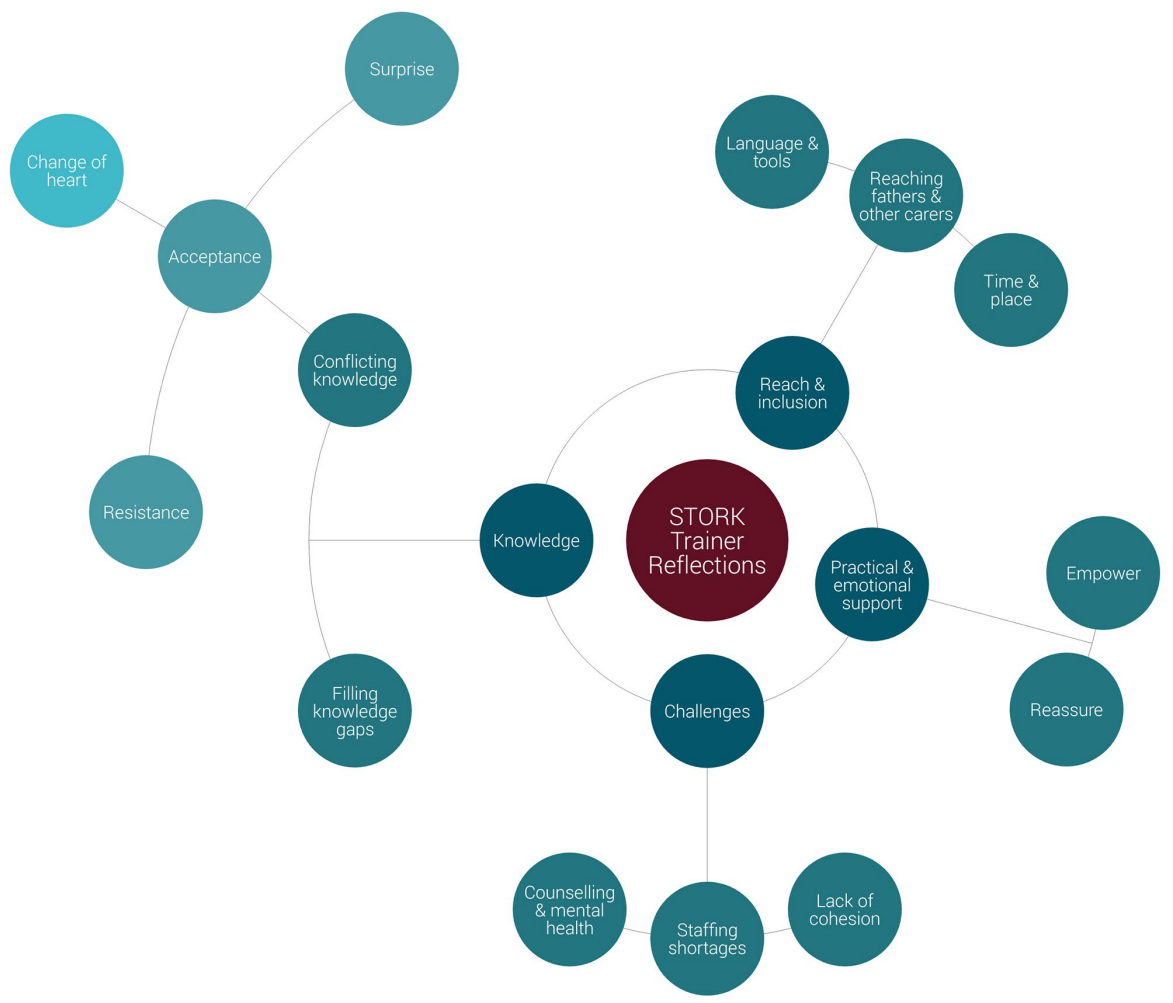
Diagrammatic representation of final themes (centre) and sub-themes (peripheral)

Second, the data were analysed for cross-cutting themes across all the categories. This stage of analysis resulted in a final list of four overall themes (reach and inclusion; knowledge; practical and emotional support; and challenges) and sub-themes, visualised in Fig. 1.

**Table 1:** Summary of initial categories and codes

Categories	Sub categories	Codes
Basic knowledge	Basic life support knowledge	Lack of knowledge - life support Previous knowledge - life support
	Breastfeeding benefits knowledge	Artificial feed misperceptions Breastfeeding - change of perception Breastfeeding - lack of knowledge (general) Challenges of knowledge gained through online groups Did not know about weaning Did not understand benefits for all babies, not only neonatal Doubts, worries and misinformation Feeding from the breast perceptions vs. expressing Knowledge gaps - when baby is hungry/full, positioning Lack of support and information on feeding from medical staff Practical and emotional support with breastfeeding/expressing
	Cultural practices, beliefs and myths	Breastfeeding cultural practice If baby gets jaundice, put them by the window Rocking baby on feet Sterilising goes against their beliefs Swaddling to straighten babies bodies Using pillow to avoid baby getting a flat head Whiskey on dummy
	ICON knowledge	Cry it out method ICON - Lack of knowledge
	Products and safety	sleep pods/nests blankets/pillows milk preparation machines
	Safe sleep knowledge	Basic safe sleep knowledge Co-sleeping Lack of information about multiple sleep options Safe sleep - lack of knowledge
	Smoking knowledge and perceptions	Did not like vaping Did not realise difference between vaping and smoking Need support to quit smoking Surprised that vaping is classed as non-smoker Understanding the importance of smoke/vape-free home
Success stories		Positive feedback/success stories Providing reassurance/refresher
Training challenges and opportunities		Additional support to quit smoking Breastfeeding – reaching mothers earlier and community follow-up Extend programme to all parents/carers beyond neonatal Improve awareness among healthcare staff of STORK Inclusion and reaching dads and other carers Language barriers - multi-language content and visual aids Need for counselling/mental health training for staff Staffing shortages and impacts of COVID-19 Unreliable Wi-Fi connection

## Results

During this time period 323 STORK programmes (i.e. distinct sessions of education with either individual parents, or in with parents and other family members such as grandparents, or carers) were offered to parents, families and carers at the Dudley Group of Hospitals NHS Trust. As this was offered during the COVID-19 period, the focus of the education and empowerment packages was heavily weighted towards parents, who had access to the hospital’s neonatal unit. A total of 524 individuals comprising 309 mothers, 198 fathers and 17 other family members including foster carers were included in the training programme during this time period.

In addition 29 staff members and students observed these sessions of education. These included including neonatal staff nurses, senior sisters, student nurses including trainee and nursing associates, doctors as well as interpreters. 12 declined the STORK programme.

The Trainers recorded 167 reflective entries during this time period. Of these, four overall themes were identified from the data: (a) reach and inclusion; (b) knowledge; (c) practical and emotional support; and (d) challenges. Each of these four themes is discussed below, covering different aspects of the STORK programme including basic life support, safe sleep, breastfeeding, smoking and infant crying. Illustrative comments were taken verbatim from the practitioner notes. The initial at the start of each quote symbolises the practitioner who made the comment (C, L or H).

### Reach and inclusion: reaching parents and carers at the right time and place, with the right tools

The degree of inclusion of different participants in the STORK programme was a theme that emerged from trainer reflections. This included the challenges of reaching fathers and other carers, as well as ‘time and place’ dimensions – reaching mothers at the right time and opportunities to extend the programme beyond the neonatal unit. The provision of multi-language and digital and audio-visual tools was proposed by the trainers to facilitate greater inclusion. The importance and challenge of engaging fathers and other carers in the programme was highlighted in the practitioner reflections. This represented a particular challenge during the COVID-19 pandemic since hospital visits were restricted, limiting opportunities to engage with fathers and wider family members.

L: ‘It was really useful to have been able to do it with the aunt, as the dad was only coming in to get the baby and would not have stayed to do STORK, meaning nobody would have had STORK if I hadn’t done it with the aunt. I think it also highlighted how STORK is not just for parents; it can be really useful for any member of the family who will be helping to look after baby. As this aunt also helped to look after other babies in the family, what she learned from STORK will not just have benefitted this baby but also probably many other babies in their family, which I feel is a really positive outcome’.

The trainers highlight the importance of reaching mothers and families at the right time, especially first-time mothers, to ensure they receive vital information on safe sleep, life support and breastfeeding. At times, trainers working in the neonatal unit reported feeling they had reached the mothers too late when providing breastfeeding support. On the other hand, due to the limited time to provide support to families in the hospital, trainers commented on the opportunity to extend support to normal care as well as into the community or establishing a STORK support network for breastfeeding mothers.

C: ‘[T]his mom said that she had never had this type of information before with any of her children as she’s normally “kicked out quickly” after birth and she’s never actively signed up to any classes. I felt that this really captured the importance of continuing with this service in the longer term so that we catch all first time moms, that way that education is following them through their subsequent babies and beyond and making differences to peoples’ lives, not in the same way as people being motivated to do it themselves as many aren’t, especially the most vulnerable’.

Language barriers were reported as a barrier to the successful delivery of the programme across all community groups. The provision of multi-language content and visual aids was mentioned by trainers as a potential solution to overcome this challenge, especially when interpreters are unavailable, and to aid parents with learning difficulties. The trainers make use of digital and audio-visual tools such as mobile apps and videos to facilitate learning. However, they encountered problems with a lack of reliable Wi-Fi connection and access to tablets in the hospital and cited a need for information leaflets in a range of languages.

### Knowledge: filling knowledge gaps and dealing with conflicting knowledge claims

The reflections revealed how the STORK programme supported parents from a range of cultural and socio-economic backgrounds, with different personal circumstances and varying levels of baseline knowledge. The provision of information tailored to the participant helped to fill knowledge gaps. When it came to safe sleep practices, ICON, smoking awareness and knowledge of basic life support for babies, the trainer reflections revealed a wide spectrum of knowledge levels among STORK participants, from no prior knowledge to some knowledge to good knowledge. Even experienced parents may have never received information on these topics and by asking questions to establish baseline knowledge, the trainers filled in gaps and provided critical information.

H: ‘When covering safe sleep and SIDs I asked the parents what they already knew, both of them had no knowledge of any safe sleep guidelines. I found this really surprising this being their 6th baby! They said that nobody had ever spoken to them about safe sleep and they had heard of cot death but associated this with babies passing away in the night, in their cots’.

C: ‘Parents appreciated everything, they came from war country and talked about war crimes and violence which they had scars… They especially appreciated the choking and resus. They said they’d never seen anything like it with the information I gave them, and took my hand in appreciation for my time to them’

The trainers described how even participants with considerable knowledge went away with new information after taking part in STORK.

C: ‘They were experienced parents, however had never seen ICON, evaluating this for the audit is a really useful way of seeing what parents’ knowledge base is and what they can gain from it. Discussed this at great length, including social isolation and coping strategies, parents took a lot from this as could understand having been in that position before. They said this was more useful than anything else in the session’.

When participants received new information that contradicted their existing knowledge, attitudes or cultural practices, this produced a range of reactions - from surprise and acceptance to resistance and dismissal. A reoccurring topic was the safety of baby products such as sleeping pods and prep machines.

L: ‘I explained to them about how (artificial formula feed) prep machines are not recommended, and explained the reasons why… They really didn’t seem very open to hearing this and I felt like everything I was saying was falling on deaf ears.… I got the impression they still fully intended on using the prep machines. This felt quite disappointing because I just couldn’t seem to get through to them’.

H: ‘After we completed the safe sleep and sudden infant death syndrome (SIDS) section mom said “Actually I get it now like why people are telling me not to do some of the stuff like putting her on her side and on the bed with pillows and stuff. I realise now that actually it’s really dangerous. I co-slept with all my other kids and used pods. Nobody has told us with our other kids not to use them and you just think stuff is safe.”’

There were cases when the trainers reported a change of heart among participants after taking part in the STORK programme. For example, mothers who had no intention to breastfeed who later decided to try it, or parents who decided not to use certain baby products after being informed about safety concerns.

L: ‘I feel quite sure that had I not intervened with STORK, this mum would never have even considered breastfeeding and no one would have encouraged her to try as whenever anyone asked she had said she was not interested. I think that everyone else involved in her care had not had the time to just sit with her for a while to talk it all through, and this is what she needed’.

### Practical and emotional support: providing reassurance and empowerment

Aside from filling knowledge gaps, the trainers described how providing practical and emotional support helped to reassure and empower parents along their journey. The trainers performed practical first aid and life support demonstrations, breastfeeding techniques, as well as offering encouragement and reassurance. Even for participants with existing knowledge of the topics covered, they reported feeling reassured and more confident after refreshing their knowledge and having the opportunity to ask questions and clarify any doubts. One mother had first-hand experience of life support since she had to previously resuscitate one of her children but was keen to do STORK for reassurance and to refresh her knowledge. The excerpts reveal the benefits of the dedicated time and counselling approach offered by trainers.

L: ‘She herself had also got a one year old child who had previously stopped breathing, and she had had to do basic life support to resuscitate them. Because of this, she was really interested in STORK, I think mostly because she wanted reassurance... For her, I think it wasn’t so much about learning from scratch, it was more about reassuring her that she would know what to do if it happened again and giving her more confidence’.

The trainers reflected on the approaches they used to empower parents. This included using a gentle approach and actively listening, especially when dealing with vulnerable participants and those that have been through complicated births.

L: ‘What I will take away from this is how important it is to just actively listen, especially when a parent is finding the neonatal unit a particularly difficult experience. I would definitely try to use this approach when speaking to parents feeling a similar way, possibly doing the goal setting with them too as this seemed to be really beneficial here and empowered her to identify her own strengths and coping mechanisms’

### Challenges for the programme

The analysis of trainer reflections highlighted a number of key challenges for the implementation of the STORK programme, including staffing shortages, the need for mental health support for trainers and lack of cohesion across healthcare staff.

Staffing shortages, compounded by additional pressures as a result of the COVID-19 pandemic, resulted in healthcare staff being overstretched and additional workload and pressure on the STORK trainers.

C: ‘Having reflected on ways to include dads in this situation, until I have support with staffing I’m finding I can’t change the situation, and it’s unfortunate people are being missed due to these barriers on the unit’.

A general lack of awareness and support on the topics covered by the STORK programme, in particular breastfeeding, among medical staff such as nurses and midwives was highlighted in the reflections. This was attributed to a lack of capacity and training and regimented feeding practices in the neonatal unit, compounded by being overstretched due to COVID-19:

C: ‘Mom really appreciated this as no feeding support given by postnatal midwives, midwives currently running at ratio 1:9 because of sickness and self-isolating due to COVID-19 and no ward breastfeeding support. This is a real barrier to parents at the moment and I feel my impact is making a real difference to these moms feeding experiences as seen here’.

This resulted in incidents of miscommunication, contradictory information being provided to patients and a general lack of coherence among STORK trainers and other staff.

C: ‘I also feel that nurses should be facilitating mom’s wishes if they ask for support to breastfeed/express/pump and should not just decide they feel mom doesn’t really want to. As our unit is not currently BFI (Breastfeeding Feeding Initiative) [accredited]. I hope that this type of training will support nurses to do this in future’.

The trainers cited difficult experiences when dealing with challenging participants and those with mental health problems. They report that previous counselling training had been beneficial and that they would have benefitted from further training in this area.

H: ‘During the session Nan started to open up about her past, being a victim of long term DV [domestic violence] herself. She expressed how hard she found it watching her daughter go through the same things. […] This situation highlighted the importance of our role not only for parents, but also the people who surround the babies we look after. It also highlights the amount of input we have not “just” delivering STORK but also providing support around SG [safeguarding], mental health and well-being’.

C: ‘On reflection I feel counselling skills are so important for this role, nearly every parent I work with has huge amounts of emotional anxiety and this has to be unpicked before learning can take place. […] The confidence and reassurance is already part of STORK, but the counselling skills and mental health support perhaps not so widely known, this is time consuming and emotionally consuming for staff, I need to ensure that I have some space to reflect as I have not been doing this recently’.

## Discussion

This novel study provides valuable insights into the current level of local understanding, limitations and challenges in key neonatal and perinatal public health messages around reducing risks for mortality in babies and infants, i.e. promoting a healthy baby and infant. We did this through trainer reflections of three STORK trainers, who had undertaken multiple STORK programme educational sessions with parents, family members and carers, with additional staff members observing. This work highlights the knowledge, successes and challenges of implementing a neonatal and infant public health STORK programme at a local district general hospital in The West Midlands. Its success with parents has been previously described from another local district hospital [5], as well as from participant evaluations, which were uniformly positive and are therefore not additionally described in this report.

Key themes and codes identified in our project (Table 1) reveal that there is still much work to be done to educate our local population around reducing risks, specifically breast feeding, safe sleeping, bystander life support, coping with a crying baby, and the risks of smoking in and after pregnancy. It is important to note that the messages we offer as part of the reducing the risk programme, are not restricted to the STORK Programme, but part of multiple NHS initiatives aimed at improving outcomes for neonates, infants and children in the UK, such as NHS Maternity Neonatal Safety Improvement Programme (promoting smoke free homes, breast feeding, early recognition of illness in baby) [10], the Maternity Transformation Programme [11], Implementing Better Births [12], The 0–19 Healthy Child Programme [13], the ICON programme, Saving Babies Lives Care Bundle [14], the NHS Long Term Care Plan [15], NHS Core20PLUS5 [16] and partly incorporated into midwifery support for families. These programmes have been in place in different formats over the years. The STORK Programme utilises these NHS resources and brings together components that target the key risks associated with infant mortality.

It is apparent from our results that despite these varied modalities of delivery to the local population, including midwifery, health visiting, neonatal, paediatric and GP services, the messages have not consistently reached the population that are childbearing and attending the local hospital’s Trust. Lack of cohesive messaging, lack of utility of modern media coupled with the pressure of work within the NHS, midwifery and health visiting sectors especially, may be responsible for this. These could partly be addressed through neonatal and perinatal public health programmes such as the STORK Programme. Such programmes are required to be expanded to the entire region, and not just to families whose babies are resident on the neonatal unit.

The value of the programme is evident in the 1:1 support, dedicated time and active listening provided to mothers and families by the STORK trainers. Utilising their previous experience in counselling, the trainers are skilled with an ability to enable, using critical thinking, emotional intelligence and thought exploration, positive experiences for the participants.

The utility of electronic mobile platforms for training health care workers in developing economies has been reviewed and has diverse potential [17]. There are no equivalent reducing the risk for infant mortality composite mobile applications reported in the literature, however the individual components in our reducing risks for infant mortality programme are key parts of many health systems internationally [19, 20] including our own [12–18], Empowering society through education and training using mobile applications, with readily available resources for parent, family or carer self-learning and updates appear to be positively received in our region with short term gains as described [5, 7]. Its benefit potentially lies in education and empowering in a multidirectional method, from parents, to grandparents and other carers of the generation before, and cascaded through discussions with friends and other family members, for the benefit of current and future generations, using modern media that is easily adopted and suited to the lifestyles of our local population. It is recognised that short term statistical reductions in infant mortality are unrealistic, considering that the associations with it span across socio-cultural, economic and generational divides. Reduction in infant mortality risk is the primary objective of the STORK programme. This perspective has been borne out in the observations from a nurse led home visiting programme, The Ohio Infant Mortality Reduction Initiative [21]. Here, this could not singlehandedly make a difference to infant mortality rates, but did contribute to prevention of some infant mortality risks.

This public health programme, utilising an easily accessible, free online mobile application unifies the public health and NHS messages important to families with a new or expectant baby and support for this is being continued through the regions’ Public Health and City Council initiatives. While further development is contingent on an effective and potentially expanded workforce, we have demonstrated through this and other local work [5, 6] that the education, empowerment and support for families to understand the risks associated with infant mortality, do not necessarily need to be delivered by highly banded nurses or doctors. Community engagement, utility of peer support workers and trainers are likely to be important in delivery of these messages, and we believe should be actively supported by local units in the region.

Infant mortality is a health inequality, as some of the risks associated with infant mortality can be linked to social inequality. Ethnicity, lack of maternal education, coupled with social deprivation mean that emphasis on providing the material in the appropriate language and at the correct pitch for families in our region is a key priority going forward [16]. Provision of appropriate resources, ensuring that the ‘reach and inclusion’ of the programme is appropriate, is critical. Utility of modern mobile applications instead of leaflets, inclusion of partners and staff in the education and empowerment package are necessary components if we are to take this public health initiative going forwards. This includes spreading the message to the population upstream of pregnancy itself. Work towards development of a senior schools programme is underway in this regard [7].

### Strengths and limitations

One of the limitations of this project is that it was mainly conducted in the post-natal period for vulnerable families and those families who had babies admitted in the neonatal unit. We believe that this work should be extended to midwifery services and provision of support in the antenatal period, the post delivery period for all women and families with a new baby and the community through engagement with all midwives, health visitors and the voluntary sector. A second limitation is that the perspectives here are solely from the trainers.

The strengths of this project are firstly that we have compiled key risks based on the associations with infant mortality in our region into an easy to use empowerment package. Secondly that we have utilised social media tools through a mobile platform, which almost all our parents have access to, and in a format that is easily understandable and quick to use for new parents who may have leaflet-fatigue. Thirdly, we have utilised reflections of key trained workers on their interactions with parents, families and carers in a free text style. This has enabled us to identify multiple, potential avenues for improvement in the programme, based on the four themes identified in the qualitative analysis. These are described in Table 2.

**Table 2:** Potential avenues for improvement

Potential Avenues for improvement	
Reach and inclusion	Devise strategies to promote greater engagement of fathers and other carers in the STORK programme.
	Consider opportunities to extend the STORK programme beyond the neonatal unit to normal care.
	Examine opportunities to reach mothers before and after childbirth, during pregnancy as well as follow-up support in the community, to maximise the impact of the programme.
	Develop training and informational content in multiple languages and formats (print, digital and audio-visual).
	Trial different approaches in training delivery (e.g. one-on-one sessions with each parent in certain situations).
	Improve access to interpreters, where possible with relevant dialects and knowledge of the content being covered in the programme.
Knowledge	Produce informational materials on topics such as baby product safety, breastfeeding and sleeping practices.
Training and capacity building	Seek measures to improve support for the STORK programme among the wider medical wards and staff, such as providing augmented breastfeeding training to midwives and nurses, including information on appropriate signposting and utility of the mobile app for this purpose.
	Provide counselling and mental health training for STORK trainers to equip them with the skills for dealing with challenging cases.

### The next steps and key challenges

Our focus now is to fine tune the programme based on potential areas for improvement that have been identified. A key priority is expanding this into a multilingual format for all to be able to use, and expand the work to evaluate birth partner perspectives. Our challenges will be how best to promote the programme and app in the antenatal period, devising strategies for roll out within the community with general practitioners, public health and health visiting as partners, to ensure that the programme is more uniformly delivered. This is important if we are to allow greater opportunity for any potential impact the programme may have in bringing about behavioural change within the community. As part of the STORK Programme in the region, our added focus must be working towards the objective of peer to peer education and ownership of the programme by the community. Enhanced training for STORK trainers to equip them with skills to deal with more complex interactions will need to be supported. In addition the study findings here provide a sound foundation for surveys and focus group discussions evaluating users’ perspectives and experiences, as well as evidence for utility and how to expand utility of the mobile app.

## Conclusion

The success of the programme is evident in the positive feedback and success stories of changes that the programme has effected in behavioural practices in its participants [7]. But behavioural adaptation as a consequence of effective education and empowerment will be the greatest challenge in defining at population level, and will require further study, followed by refinement of messaging and further study over time. All of this is contingent on getting the messaging, its delivery, reach and inclusion appropriate for the local population.

## Data Availability

The data set used to perform the qualitative analysis can be requested through contacting the corresponding author. They are not made publicly available as they are pseudo-anonymised reflections of key personnel conducting the STORK programme at the hospital.
